# Epigenetic instability of imprinted genes in human cancers

**DOI:** 10.1093/nar/gkv867

**Published:** 2015-09-03

**Authors:** Joomyeong Kim, Corey L. Bretz, Suman Lee

**Affiliations:** Department of Biological Sciences, Louisiana State University, Baton Rouge, LA 70803, USA

## Abstract

Many imprinted genes are often epigenetically affected in human cancers due to their functional linkage to insulin and insulin-like growth factor signaling pathways. Thus, the current study systematically characterized the epigenetic instability of imprinted genes in multiple human cancers. First, the survey results from TCGA (The Cancer Genome Atlas) revealed that the expression levels of the majority of imprinted genes are downregulated in primary tumors compared to normal cells. These changes are also accompanied by DNA methylation level changes in several imprinted domains, such as the *PEG3*, *MEST* and *GNAS* domains. Second, these DNA methylation level changes were further confirmed manually using several sets of cancer DNA. According to the results, the Imprinting Control Regions of the *PEG3*, *MEST* and *GNAS* domains are indeed affected in breast, lung and ovarian cancers. This DNA methylation survey also revealed that evolutionarily conserved *cis*-regulatory elements within these imprinted domains are very variable in both normal and cancer cells. Overall, this study highlights the epigenetic instability of imprinted domains in human cancers and further suggests its potential use as cancer biomarkers.

## INTRODUCTION

In mammals, a subset of autosomal genes are subject to an unusual dosage control mechanism termed genomic imprinting, by which only one allele for a given gene is expressed and functional. The majority of imprinted genes play critical roles in controlling fetal growth rates (1). Consistent with this, the biochemical functions of imprinted genes tend to be clustered in insulin or insulin-like growth factor signaling pathways. As a consequence, imprinted genes have been quite often identified as the genes affected in human cancers, highlighting the close linkage of imprinted genes to growth factor signaling pathways (2,3). Among the 100 imprinted genes found in the human genome, the well-known examples include *H19, IGF2* (Insulin-like growth factor 2), *IGF2R* (IGF2 receptor), *GNAS* (stimulatory GTPase α), *GRB10* (Growth factor-bound protein 10), *MEST* (Mesoderm-Specific Transcript) and *PEG3* (Paternally Expressed Gene 3) (3).

Imprinted genes are also clustered in specific chromosomal domains, size-ranging from 0.5 to 2-megabase pair in length. Yet, small genomic regions, 2–4 kb in length, are known to control the imprinting of large genomic domains, thus named Imprinting Control Regions (ICRs) (4). One of the main functions of ICRs is first to inherit germ cell-driven DNA methylation as a gametic signal, and later to maintain the subsequent allele-specific DNA methylation pattern within somatic cells (5). Any slight change in the DNA methylation level of an ICR usually causes global and catastrophic outcomes in the imprinting of the corresponding domain, underscoring the significant roles played by ICRs (4–7). Nevertheless, the DNA methylation levels of ICRs are believed to be very vulnerable to genetic and epigenetic changes due to the opposite functional needs required by the two parental alleles within the same somatic cell: one attracting versus the other repelling DNA methylation. Given this unusual property, ICRs may be one of the most epigenetically unstable regions in the human genome during tumorigenesis. However, the epigenetic instability at ICRs has never been tested in the context of tumorigenesis, thus how early and how often the DNA methylation levels of ICRs are affected in what types of cancers is currently unknown.

In the current study, therefore, we conducted a series of experiments to characterize the epigenetic instability of ICRs and other regulatory regions within imprinted domains. According to the results, the expression and DNA methylation levels of the majority of imprinted domains are often affected in various human cancers. Several series of DNA methylation analyses confirmed that the ICRs of *PEG3*, *MEST* and *GNAS* domains are frequently affected in various human cancers. Also, the DNA methylation levels of several evolutionarily conserved regions within these imprinted domains, potential enhancers, are also variable in both normal and cancer cells. Overall, these results demonstrated that the DNA methylation status of imprinted domains is quite unstable in various human cancers.

## MATERIALS AND METHODS

### Expression and DNA methylation survey using TCGA

**Figure 1.**
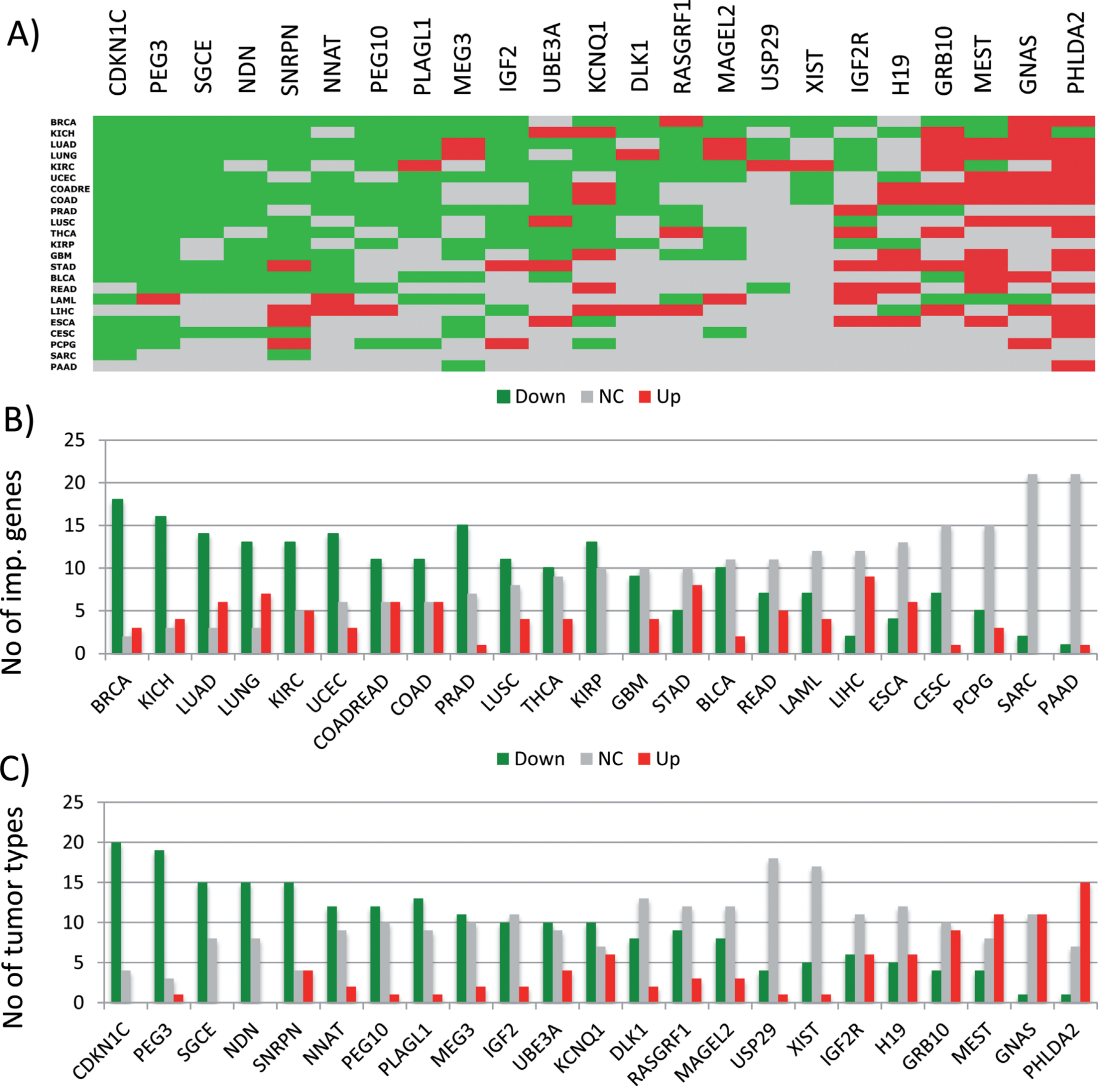
Expression level changes of imprinted genes in human cancers. (**A**) Expression levels of imprinted genes (represented by columns) were compared between the primary tumor and normal solid tissue cells of various cancers (represented by rows) using TCGA database (The Cancer Genome Atlas). Statistically significant down- and upregulations were indicated by green and red boxes, respectively, while no significant change with gray boxes. *XIST* has been included in this series of analyses due to its 50% DNA methylation levels in females although it is not an imprinted gene. The actual table used for this image is available as Supplementary Data 1. (**B**) This graph summarizes how many imprinted genes show down and upregulations for each cancer type. (**C**) This graph summarizes in how many cancer types the expression levels of each imprinted gene are changed between the tumor and normal cells. Individual cancer types are represented with the following abbreviations: BRCA (breast invasive carcinoma), KICH (kidney chromophobe), LUAD (lung adenocarcinoma), LUNG (lung cancer), KIRC (kidney renal clear cell carcinoma), UCEC (uterine corpus endometrioid carcinoma), COADREAD (colon and rectum adenocarcinoma), COAD (colon adenocarcinoma), PRAD (prostate adenocarcinoma), LUSC (lung squamous cell carcinoma), THCA (thyroid carcinoma), KIRP (kidney renal papillary cell carcinoma), GBM (glioblastoma multiforme), STAD (stomach adenocarcinoma), BLCA (bladder urothelial carcinoma), READ (rectum adenocarcinoma), LAML (acute myeloid leukemia), LIHC (liver hepatocellular carcinoma), ESCA (esophageal carcinoma), CESC (cervical squamous cell carcinoma and endocervical adenocarcinoma), PCPG (pheochromocytoma and paraganglioma), SARC (sarcoma) and PAAD (pancreatic adenocarcinoma).

**Figure 2.**
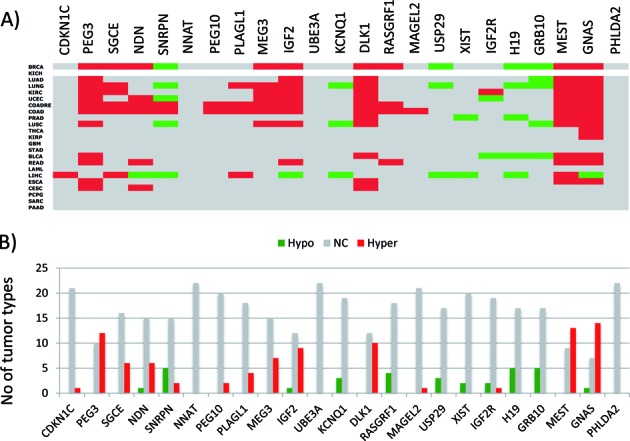
DNA methylation level changes of imprinted genes in human cancers. (**A**) DNA methylation levels of imprinted genes (represented by columns) were compared between the primary tumor and normal solid tissue cells of various cancers (represented by rows) using TCGA database (The Cancer Genome Atlas). Statistically significant down- and upregulations were indicated by green and red boxes, respectively, while no significant change with gray boxes. The actual table used for this image is available as Supplementary Data 2. (**B**) This graph summarizes in how many cancer types the DNA methylation levels of each imprinted gene differ between the tumor and normal cells.

**Figure 3.**
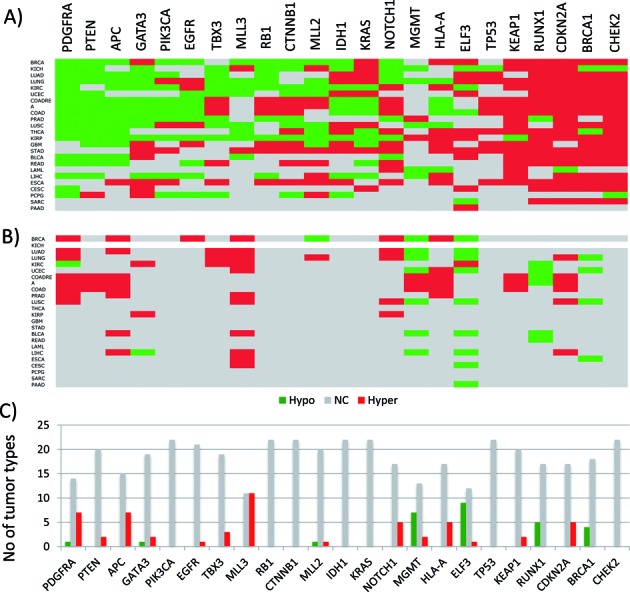
Expression and DNA methylation level changes of cancer genes. Expression levels (**A**) and DNA methylation levels (**B**) of cancer genes (represented by columns) were compared between the primary tumor and normal solid tissue cells of various cancers (represented by rows) using TCGA database (The Cancer Genome Atlas). Statistically significant down- and upregulations were indicated by green and red boxes, respectively, while no significant change with gray boxes. The actual tables used for these images are available as Supplementary Data 3–4. (**C**) This graph summarizes in how many cancer types the DNA methylation levels of each gene are different between the tumor and normal cells.

The expression and DNA methylation levels of individual genes were compared between the primary tumor and normal solid cells using the data sets available through TCGA (https://genome-cancer.ucsc.edu/proj/site/hgHeatmap/). The gene expression set (IlluminaHiSeq percentile) from each of 23 individual cancer types was used for analyzing the expression level changes, while the DNA methylation set (Methylation 450K) from each of 22 cancer types was used for analyzing the DNA methylation changes for each gene. Student *t*-tests were performed to determine the statistical significance of each observed change. The default *P*-value in TCGA was set at <0.05. Statistically significant changes were scored and summarized using several tables and graphs (Figures 1–3 and Supplementary Data 1–4).

### DNA methylation analyses by COBRA and individual sequencing

The current study used the following two sets of DNA that were obtained from a commercial firm (BioChain). The first set contains DNA from two normal tissues (brain, lot# A712209; liver, lot# A908154) and six cancer tissues (breast, lot# A805125; lung, lot# B702148; kidney, lot# B301004; colon, lot# B410199; ovary, lot# A901085; liver, lot# A908154). Two additional sets of matched pairs have been later included as the first set of DNA (breast, lot#B412015; lung, lot# A811204). The second set contains DNA from 8 normal and 40 breast cancer tissues (Cat. No. D8235086–1). Each of these DNA (1 μg) was treated with the bisulfite conversion protocol using a commercial kit (EZ DNA methylation kit, Zymo Research). The converted DNA was used for polymerase chain reaction (PCR) amplification. The detailed information regarding the sequences and genomic position for each oligonucleotide set is available in Supplementary Data 5. The amplified PCR products from the bisulfite-converted DNA were digested with restriction enzymes, separated on a 2% agarose gel and the relative amount of each digested DNA fragment was measured based on its band density as described below. Quantity One software was used to export gel electrophoresis images as lossless tiff files (Gel Doc system, BioRad). Tiff files were then processed as 8-bit grayscale using ImageJ software (8) in the following manner: (i) data was inverted; (ii) background was subtracted using default setting; (iii) brightness/contrast was adjusted by selecting the auto adjust command one time; (iv) bands in each lane were individually selected using the rectangular tool; (v) density plots were then generated for each rectangular selection; (vi) each density peak was gated at the base of the peak at a location higher than background signals using the line drawing tool; and (vii) the area under each peak was automatically generated by the software using the wand tool. Area results were exported into an Excel spreadsheet where all subsequent analyses were performed. DNA methylation values (%) were calculated using the following formula: 100*((area of peak from bands indicating unmethylation)/(area of peak from bands indicating methylation + area of peak from bands indicating unmethylation)). ANOVA single factor statistical analysis was performed on the percent methylation results for each locus screened. If the *P*-value from the ANOVA analysis was ≤0.05, then subsequent pairwise *t*-test (two sample assuming equal variance) was performed comparing each tumor sample to each normal tissue for each locus. Three independent trials starting from bisulfite conversion to restriction digestion followed by densitometry were repeated to derive the average DNA methylation levels of each locus with 95% confidence intervals.

The PCR products amplified from the first set of bisulfite-converted DNA were also sequenced with the following strategy. The 144 PCR products (8 tissues × 16 genomic regions) were grouped together, end-repaired and ligated with two duplex adaptors, Ion Torrent P1 and A adaptors, with the following kit supplemented with T4 ligase and Bst 2.0 WarmStart DNA polymerase (NEBNext End Repair Module, New England Biolabs, Cat. No. E6050S). The pool of ligated PCR products was subsequently separated with agarose gel electrophoresis and used for isolating the DNA fragments size-ranging from 200 to 400 bp in length. The library of isolated DNA fragments was amplified with the following two primers: 5′-CCACTACGCCTCCGCTTTCCTCT-3′ and 5′-CCATCTCATCCCTGCGTGTCTCC-3′ corresponding to the two adaptors. The amplified library was further processed for sequencing on a next-generation-sequencing (NGS) platform (PGM2, Ion Torrent, Life Technologies). The raw sequence reads were processed in the following manner. The sequence reads smaller than 100 bp in length were removed, and the remaining sequences were sorted to an initial PCR product based on its two primer sequences. The sorted sequences for each locus were used for calculating DNA methylation levels for each locus using BiQ Analyzer HT tool (9). The bioinformatic pipeline used for this process is available upon request.

## RESULTS

### Expression level changes of imprinted genes in human cancers

In the current study, TCGA (The Cancer Genome Atlas) database was used to survey the expression and DNA methylation level changes of imprinted genes between primary tumor and normal tissues (10). Out of 33 different cancer types, 23 individual cancers have two datasets, gene expression and DNA methylation, thus these were used for analyzing 23 different imprinted genes (Figures 1 and 2). The expression levels of each imprinted gene were first compared between the primary tumor and normal solid tissue cells for each cancer type. Later, the statistical significance of the observed change was determined using Student's *t*-test. This large set of surveys (representing total 529 individual cases analyzing 23 imprinted genes in 23 different cancers) has been summarized in the following manner (Figure 1). For each gene, statistically significant changes, either down- or upregulation in a given cancer type, were presented in green and red color boxes, respectively, while no significant changes in gray boxes. The order of rows from top to bottom (representing different cancer types) indicates which cancer type shows from the greatest to smallest numbers of the imprinted genes with the expression level changes between the tumor and normal cells. On the other hand, the order of columns from left to right (representing individual imprinted genes) indicates the degree of tendency to which individual imprinted genes are prone to be from down- to upregulated in various cancer types.

According to the results, the expression levels of imprinted genes are differentially affected among individual cancer types (Figure 1B). For instance, the majority of imprinted genes tend to be affected in the following cancer types: BRCA (breast invasive cancer, 21 out of 23 genes), KICH (kidney chromophobe, 20/23), LUAD (lung adenocarcinoma, 20/23). In contrast, a very few imprinted genes are affected in the following cancers: SARC (sarcoma, 2/23) and PAAD (pancreatic adenocarcinoma, 2/23). Also, imprinted genes tend to be more downregulated than upregulated in the primary tumors, showing 217 downregulations with green boxes versus 91 upregulations with red boxes in the entire set of 529 cases. The significance of this observed trend was further tested through performing a similar series of analyses with a gene set in TCGA, which are known for their frequent mutations in various human cancers (named PANCAN in Figure 3A). In this set of cancer genes, the numbers of the down- and upregulated cases are similar to each other with 159 versus 184 cases in the entire set of 529 cases, which is significantly different from the pattern observed in the imprinted gene set (*P* < 0.0001, Fisher's exact test). This suggests that imprinted genes may contribute to tumorigenesis more as a tumor suppressor than as an oncogene. Among the imprinted genes, two genes (*CDKN1C* and *PEG3*) are most frequently downregulated, showing the downregulation in 20 and 19 out of 23 different caner types, respectively (Figure 1C). In contrast, the following three genes are frequently upregulated in the primary tumors, including *MEST* (11/23), *GNAS* (11/23) and *PHLDA2* (15/23). In summary, this series of analyses indicated that breast (BRCA), kidney (KICH) and lung (LUAD) cancers are the most frequent cancer types displaying the expression level change of imprinted genes, and that imprinted genes tend to be more downregulated than upregulated in human cancers.

### DNA methylation level changes of imprinted genes in human cancers

DNA methylation level differences of each imprinted gene between the primary tumor and normal cells were also analyzed using the dataset of TCGA. The DNA methylation data in TCGA have been derived from hybridization-based experiments surveying the 450 000 CpG sites that are spread throughout the human genome (11). Thus, a given genomic interval usually has several CpG sites that are derived from the different part of a gene, such as promoters, enhancers and exons, with different levels of DNA methylation depending on their functional roles. For this series of analyses, however, we mainly focused on the methylation levels of the CpG sites that are located within the promoters and ICRs of each imprinted domain. In most cases, the genomic regions surrounding transcription start sites were regarded as the promoters for imprinted genes. In the case of ICRs, the sequence and position information of mouse imprinted domains were used as a guide for identifying human counterparts. The genomic coordinates for each imprinted domain used for this manual inspection are available in Supplementary Data 6.

The DNA methylation levels of 23 imprinted genes were first compared between the tumor and normal tissues for each of 22 individual cancer types with a similar scheme as the expression dataset (Figure 2). Statistically significant changes were determined and summarized in an identically formatted table as the expression dataset with the same orders of rows (cancer types) and columns (imprinted genes) (Figure 2A). The dataset from KICH (kidney chromophobe), however, does not have two clear groups, tumor and normal cells, thus omitted in this series of analyses (empty row in Figure 2A). Inspection of the summary table provides the following immediate conclusions. First, the number of cases showing DNA methylation changes (120/504) is far less than that of the expression dataset (308/529), suggesting that DNA methylation change is a much rare event than expression level change in cancers. Yet, this number is still greater than the total number of cases observed from the PANCAN set (82/504), indicating relatively high levels of epigenetic instability among the imprinted genes in human cancers (Figure 2B). Second, the number of the imprinted genes with hypermethylation (marked in red) is much greater than that with hypomethylation (marked in green), showing 88 versus 32 cases, respectively. This is consistent with the earlier observation that the functional contribution of imprinted genes to human cancers is more closely associated with tumor suppressor roles (Figure 1). Third, the DNA methylation changes appear to occur more frequently in a small subset of imprinted genes, including *PEG3* (12/22), *IGF2* (9/22), *DLK1* (10/22), *MEST* (13/22) and *GNAS* (15/22) (*P* < 0.0001, χ2 test). This indicates that some imprinted genes are more unstable, epigenetically, than the others. Also, the direction of changes in these genes is mostly hypermethylation, although there are some genes with hypomethylation in cancers, such as *SNRPN* (5/22) and *H19* (5/22). Nevertheless, the total number of instances of hypomethylation is significantly less than those of hypermethylation (*P* < 0.0001, χ2 test). It is important to note that two upregulated genes, *GNAS* and *MEST*, are also hypermethylated. Detailed inspection revealed that the ICRs of these two genes are in fact the promoters of the antisense genes for both cases. Thus, the DNA hypermethylation on these two antisense genes are likely responsible for the upregulation of the corresponding sense genes. Interestingly, a small subset of genes are also accountable for the majority of observed DNA methylation changes among the PANCAN set, such as *APC* (7/22), *MLL3* (11/22), *MGMT* (10/22) and *ELF3* (10/22) (*P* < 0.0001, χ2 test) (Figure 3B and C). This indicates that a subset of the cancer gene set is also more unstable than the others. In this case, two genes (*APC* and *MLL3*) are hypermethylated whereas the other two (*MGMT* and *ELF3*) are hypomethylated. In summary, a series of DNA methylation surveys revealed that a relatively large fraction of imprinted genes are epigenetically affected in human cancers, and also that this epigenetic instability is particularly pronounced in a subset of imprinted genes, such as *PEG3*, *IGF2*, *DLK1*, *MEST* and *GNAS*.

### DNA methylation levels of imprinting control regions in human cancers

**Figure 4.**
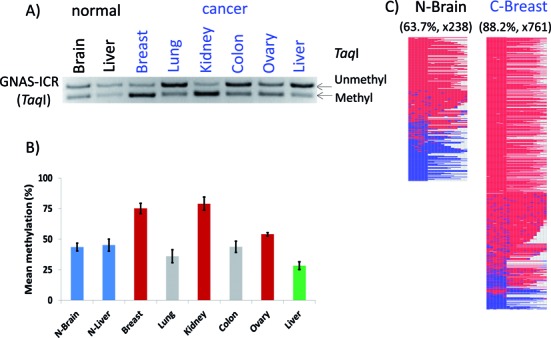
DNA methylation analysis of GNAS-ICR. A set of human DNA derived from two normal (brain and liver) and six cancer tissues (breast, lung, kidney, colon, ovary and liver) were treated with the bisulfite conversion protocol, which were then analyzed with COBRA (**A**) and individual sequencing (**C**). For COBRA analyses, the PCR products from GNAS-ICR were digested with TaqI, displaying the unmethylated and methylated portion of each PCR product. The amounts of these two fragments from three independent trials were quantified based on their band density using ImageJ software, and mean methylation with 95% confidence intervals were plotted (**B**). As predicted, two normal tissues displayed 50% methylation levels on GNAS-ICR. On the other hand, three cancer tissues showed hypermethylation and one tissue displayed hypomethylation. All of these PCR products were also sequenced using a NGS platform, and the bisulfite sequence reads were analyzed with BiQ Analyzer HT Tool (C). Two representative graphic illustrations are shown: red and blue boxes indicate methylated and unmethylated CpG sites, respectively. The names of some of tissues start with either N- or C- to differentiate the origin of samples, normal and cancer tissues.

The DNA methylation changes of imprinted genes observed in human cancers were further characterized with the following scheme. First, we targeted the following loci given the initial observation from TCGA: five imprinted domains (*PEG3*, *H19*/*IGF2*, *DLK1*/*GTL2*, *MEST*, *GNAS*) and four cancer genes (*APC*, *MLL3*, *MGMT*, *ELF3*). Second, we used a DNA panel that is comprised of two normal tissues (brain and liver) and six cancer tissues (breast, lung, kidney, colon, ovary, liver) (Figure 4A). This set of DNA panel also included one matched pair with two liver samples from the same individual. Later, we have included two additional sets of matched pairs for breast and lung cancers given the anticipated epigenetic heterogeneity between different individuals, (Supplementary Data 8). Third, we used the bisulfite conversion protocol for DNA methylation analyses. The converted DNA was amplified with PCR, which was then analyzed first by COBRA (COmbine Bisulfite Restriction Analysis; 12) and later by individual sequencing on an NGS platform (Figure 4A and C). Any potential change detected through one approach was carefully reanalyzed through the other method.

The results from the *GNAS* locus have been presented as a representative set (Figure 4). In terms of nomenclature, a genomic region covering an ICR is named with a locus name followed by—ICR, for instance GNAS-ICR. On the other hand, the promoter region of an imprinted gene is named with the locus name followed by—Pro. The DNA methylation level of GNAS-ICR is thought to be 50% in any given normal tissue based on the allele-specific methylation pattern, which is established during gametogenesis and subsequently inherited as a gametic signal. The methylation level of ICRs (50%) is also tightly controlled in somatic cells (1,4). Indeed, this turned out to be the case: the two normal tissues displayed an equal amount of the two DNA fragments representing the methylated and unmethylated DNA for this ICR (brain and liver in lane 1 and 2 on Figure 4A). By contrast, the other six products from cancer samples displayed either hyper or hypomethylation as compared to the levels of the two normal tissues: hypermethylation in breast, kidney and ovarian cancers versus hypomethylation in liver cancer. This series of COBRA analyses were repeated three times from bisulfite conversion to restriction enzyme digestion, thus rendering statistical tests for each observed pattern. According to the results from the statistical tests, GNAS-ICR was found to be hypermethylated in breast, kidney and ovarian cancers, whereas the same ICR was hypomethylated in liver cancer (Figure 4B). Since COBRA measures DNA methylation at only one CpG site, we also sequenced the PCR products that had been used for COBRA to further confirm the initial observations (Figure 4C). The results from sequencing also supported the majority of the initial observations, showing higher DNA methylation levels in the breast, kidney and ovarian cancers (Figure 4C and Supplementary Data 9c). However, the hypomethylation observed in liver was not confirmed through the sequencing method, thus this observation is regarded as inconclusive (Supplementary Data 9c).

**Figure 5.**
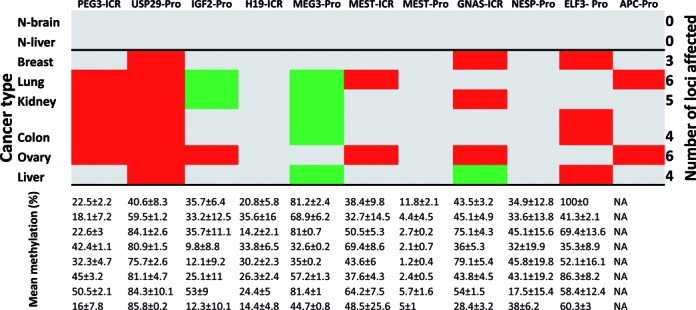
Summary of DNA methylation level changes. A series of DNA methylation analyses on imprinted genes were conducted using a set of normal and cancer samples. The results were subsequently summarized with a table showing the average DNA methylation level and corresponding 95% confidence interval per each sample in a given tissue (bottom). Statistically significant changes relative to those observed from two normal tissues determined by *t*-test were also summarized in a separate table (top). In this table, red, green and gray boxes indicate hyper, hypomethylation and no change, respectively. It is important to note that some of ICRs and DMRs did not show 50% DNA methylation levels in normal tissues due to the technical issues that are mainly caused by inefficient restriction enzyme digestion.

All the other remaining regions, totaling 16 genomic regions, were also analyzed in a similar manner, thus providing DNA methylation levels for each locus in each DNA sample of this cancer DNA panel (Figure 5 and Supplementary Data 7–9). Among the five imprinted domains, however, the results from *DLK1* in the *DLK1*/*MEG3* domain are missing due to the technical difficulties associated with PCR and other steps. It is also important to note that although all the ICRs are supposed to show 50% methylation levels the actual values obtained through the two methods are sometimes variable due to technical caveats, such as inefficient restriction enzyme digestion in the case of COBRA and also preferential amplification of methylated DNA in the case of NGS-based sequencing (13). Nevertheless, tabulation and examination of the results provide the following conclusions. First, the overall pattern from this survey is mostly consistent with the pattern from TCGA although there are some discrepancies. For instance, *IGF2* appears to be hypomethylated in this survey although it was initially identified as the hypermethylated gene in TCGA (Figure 2). Conversely, *USP29* is found to be hypermethylated in the majority of the tested samples although this locus was initially identified as a hypomethylated gene only in a small subset of cancer types in TCGA (3/23 in Figure 2). One of the main culprits for these discrepancies may be related to the insufficient coverage of CpG sites for a given gene's promoter in TCGA. In many cases, the density of CpG sites in Human450K array is usually low such that the information from TCGA usually needs to be further verified by individual experiments. Second, the most consistent pattern between the two surveys was observed from the DNA methylation levels of ICRs, in particular the ICRs of the *PEG3*, *MEST* and *GNAS* domains. These three ICRs were previously identified as hypermethylated regions from TCGA (Figure 2), which was again confirmed through individual DNA methylation surveys in this study (Figure 5 and Supplementary Data 8). Furthermore, it is also important to point out the fact that the survey results from TCGA can be readily demonstrated through a small-size cancer DNA panel, including three sets of matched pair samples, highlighting the point that ICRs are indeed unstable epigenetically at relatively high frequencies in human cancers.

### DNA methylation levels of *cis*-regulatory elements in human cancers

**Figure 6.**
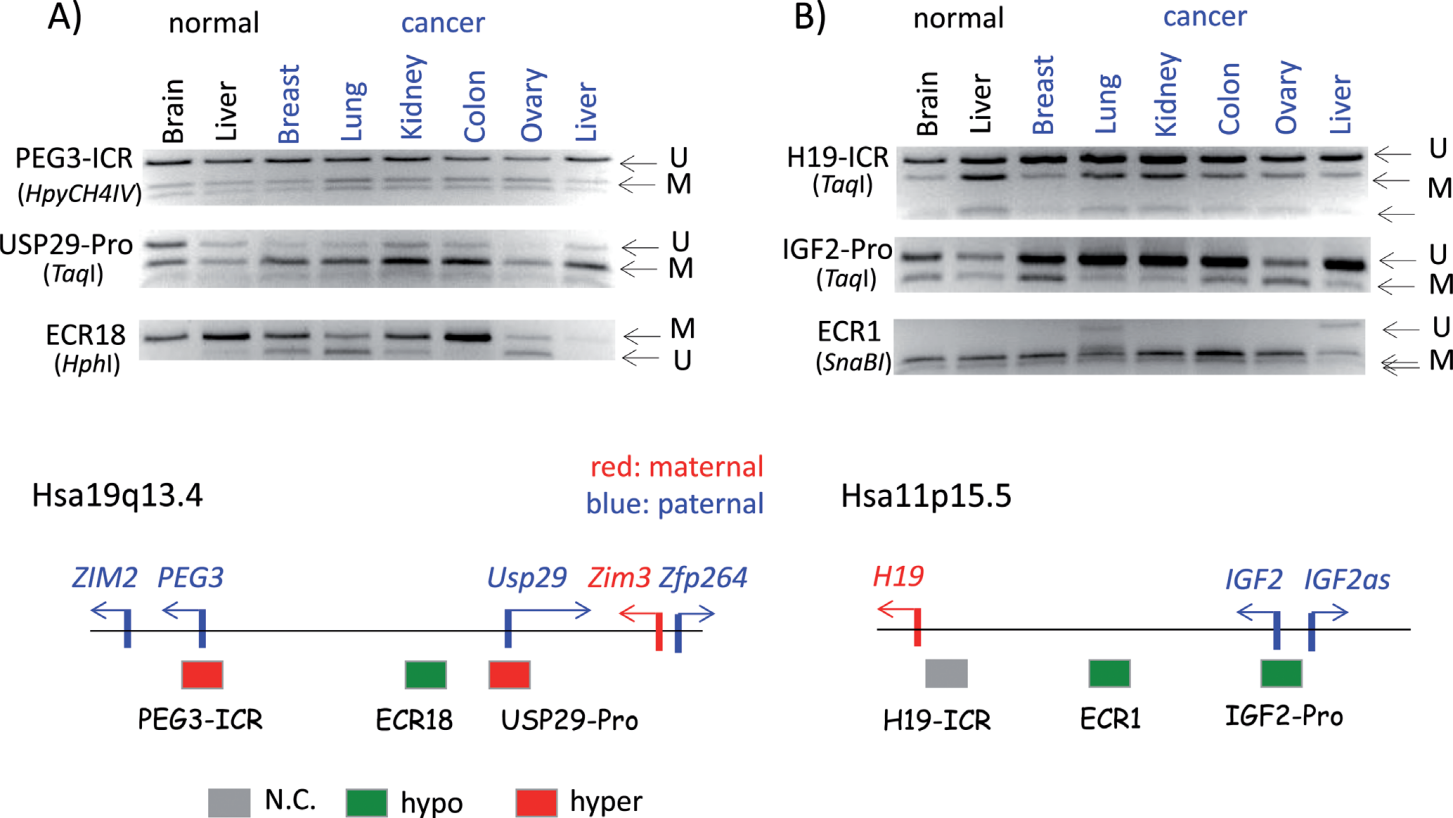
DNA methylation level changes of the *PEG3* and *H19*/*IGF2* domains. A set of human DNA derived from two normal (brain and liver) and six cancer tissues (breast, lung, kidney, colon, ovary and liver) were used to survey the DNA methylation levels of the *PEG3* domain (**A**) and the *H19*/*IGF2* domain (**B**). Three genomic regions for each domain were targeted and analyzed with COBRA as shown in the upper panel. The bottom panel shows the genomic structures of two imprinted domains along with their DNA methylation changes observed in cancers.

DNA methylation studies have traditionally focused on CpG-rich regions, such as promoters. This is also the case for the Human450K array, the survey platform used for TCGA, in which the majority of CpG sites are within genes’ promoters (11). According to recent results, however, more dynamic changes in DNA methylation levels are usually detected in the enhancer regions than in the promoter regions of mammalian genes (14,15). In that regard, it is also relevant to point out that the function of each imprinted domains is usually regulated through long-distance *cis*-regulatory elements, potential enhancers or insulators (1,4). Thus, we have included a couple of these *cis*-regulatory elements in this series of DNA methylation analyses (Figure 6). First, the *PEG3* domain is represented by two main genes, *PEG3* and *USP29*, which are separated by a 250-kb genomic region. This 250-kb genomic region contains many evolutionarily conserved regions, and one of these, called ECR18 (Evolutionarily Conserved Region 18), is known to function as an enhancer for several promoters within this domains (16). The DNA methylation levels of ECR18 were measured and compared with those from the promoter regions of *PEG3* and *USP29*. The promoter of *PEG3* overlaps with the ICR of this domain, thus named PEG3-ICR. The two promoter regions are hypermethylated in several cancer samples, yet the DNA methylation levels of ECR18 appear to become hypomethylated in a similar set of cancer samples (Figure 6A and Supplementary Data 8). The genomic distance between ECR18 and USP29-Pro is only about 30 kb in length, yet the DNA methylation level changes of the two regions become opposite in the cancer samples: one with hypermethylation and the other hypomethylation. A similar Evolutionarily Conserved Region (ECR) is also localized within the *H19*/*IGF2* domain, thus named arbitrarily ECR1 (previously named Centrally Conserved Domain; 17). This region is predicted to be a potential enhancer given the previous studies as well as the histone modification profiles associated with this region, showing high levels of H3K27ac in various somatic tissues in both human and mouse (18). DNA methylation analyses also revealed a similar conclusion as seen in the ECR18 of the PEG3 domain. The ECR1 of the *H19*/*IGF2* domain is usually methylated in normal tissues, but it becomes hypomethylated in a couple of cancer samples, including lung and liver cancers (Figure 6B). It is interesting to point out that both ECRs seem to lose DNA methylation in cancer samples. Overall, this series of DNA methylation surveys revealed that the DNA methylation levels of ECRs within imprinted domains are variable among and between tumor and normal cells.

### Frequency of DNA methylation change of imprinted genes in human cancers

**Figure 7.**
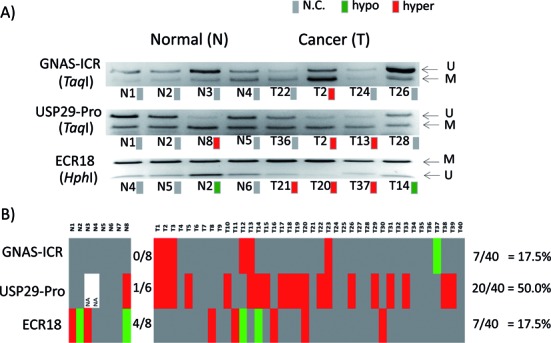
Frequency of DNA methylation changes among the normal and cancer samples. The DNA methylation changes observed in three genomic regions (GNAS-ICR, USP29-Pro, ECR18) were further analyzed in terms of their frequency using a set of breast DNA, which is made of 8 normal and 40 cancer samples. A subset of COBRA results from three regions is presented (**A**), and the entire set of results is also summarized as a table (**B**).

The DNA methylation level changes observed in human cancers were also analyzed in terms of their frequency among human cancers. For this series of analyses, we used a panel of breast cancer DNA that is composed of 8 normal and 40 cancer samples. Since the amount of each sample is very limited, three genomic regions were analyzed with COBRA (Figure 7). According to the results, the DNA methylation level of GNAS-ICR did not show any changes among the 8 normal samples, but it did show 7 changes out of the 40 tested cancer samples, resulting in the frequency of 17.5% among the breast cancer set. In the case of USP29-Pro, changes in the DNA methylation occurred in 1 out of 6 normal and 20 out of 40 cancer samples, resulting in 16.6 and 50% frequencies in the normal and cancer sets, respectively. The frequency of USP29-Pro (50%) is much higher than that of GNAS-ICR (17.5%), but the methylation change associated with *USP29* may need more caution than that of GNAS-ICR since one of the normal samples also showed DNA methylation change. This suggests that USP29-Pro might be more variable than GNAS-ICR among the normal samples. This also turns out to be the case for the ECR18 of the *PEG3* domain. The DNA methylation levels of ECR18 are very variable among the normal and cancer samples, showing 4/8 and 7/40, respectively. Taken together, the DNA methylation levels of GNAS-ICR do not show any variation among the normal samples, but show some levels of changes among the cancer samples. In contrast, the DNA methylation levels of the other regions, such as USP29-Pro and ECR18, were shown to be more fluctuating in both normal and cancer samples. This pattern may be reflecting the fact that the DNA methylation levels of an ICR are more tightly controlled than the other regions due to its significant roles in regulating the function of the imprinted domain.

## DISCUSSION

In the current study, we have characterized the epigenetic instability of imprinted genes in human cancers using two different approaches. First, the expression and DNA methylation levels of imprinted genes were compared between tumor and normal cells using the TCGA database. Second, a set of imprinted genes was targeted and further characterized with DNA methylation analyses using two sets of cancer DNA panels. The results from these approaches confirmed that the DNA methylation levels of imprinted genes, particularly ICRs, are indeed very unstable in human cancers.

According to the survey results from TCGA, the expression levels of the majority of imprinted genes tend to be downregulated in concert with DNA hypermethylation on their promoter and ICRs (Figures 1 and 2). The observed patterns from imprinted genes are interesting for the following reasons. First, the number of downregulations is much greater than that of upregulations among imprinted genes, 217 versus 91 cases (Figure 1). This is significantly different from the down- and upregulated cases observed from the PANCAN set, 159 versus 184 cases (Figure 3). This suggests that the function of many imprinted genes may be more closely aligned with tumor suppressors than oncogenes in human cancers. It has also been previously hypothesized that paternally and maternally expressed genes might function as an oncogene and tumor suppressor, respectively (19). According to a separate inspection, however, we did not find any strong indication supporting this possibility that the down- or upregulated genes have any bias toward one parental allele. Second, the number of the DNA methylation changes associated with imprinted genes is quite different from that of the PANCAN set, which are non-imprinted but cancer-related (Figures 2 and 3). The total number of changes (120) is greater than that of the PANCAN set (82), suggesting that the DNA methylation status of imprinted genes is more vulnerable than that of these cancer-related genes. In imprinted genes, one allele is already inactivated by DNA methylation as a naturally occurring ‘one hit’ such that DNA methylation on the remaining active alleles should have an immediate impact on the functions of the genes in cancer cells. This might explain the higher degree of epigenetic instability observed from the imprinted gene set. Also, the direction of DNA methylation changes is skewed more toward hypermethylation than hypomethylation (88 versus 32). This is quite intriguing given the normal 50% DNA methylation levels of imprinted genes, which have an equal probability of becoming hyper or hypomethylated by either gaining or losing DNA methylation in cancer cells. Nevertheless, imprinted genes are shown to be more hypermethylated than hypomethylated. Overall, this agrees with the pattern observed from the expression level changes showing more downregulation than upregulation since DNA methylation is regarded as a repressor for gene expression (20,21).

The survey results from DNA methylation also provide an interesting aspect of imprinted genes. The DNA methylation change occurs more frequent in a subset of imprinted genes, such as *PEG3*, *DLK1*, *MEST* and *GNAS* (Figures 2 and 5). More importantly, the genomic regions associated with these imprinted genes are all ICRs except *DLK1*. There are two possible scenarios that may be responsible for this epigenetic instability associated with ICRs. First, the epigenetic instability associated with ICRs may be an outcome of functional selection of the corresponding imprinted domains, resulting in a gain of fitness over normal cell counterparts. One of main functions of ICRs is controlling the mono-allelic expression (or gene dosage) of imprinted genes in a given domain. Potential malfunctions of ICRs caused by their abnormal DNA methylation levels, either hyper or hypomethylation, could easily disrupt the fine-tuned gene dosage of individual imprinted genes, eventually helping tumorigenesis. Second, the epigenetic instability may be simply reflecting the unusual properties associated with the genomic sequences of ICRs. As described earlier, the genomic sequences of ICRs contain two conflicting features: one allele attracting versus the other repelling DNA methylation. As a consequence, the ICRs often contain CpG-rich sequences that are usually protected from DNA methylation. At the same time, they also contain tandem repeat sequences that tend to attract the repression marks, such as H3K9me3 and DNA methylation (22). In cancer cells, these two conflicting features might make ICRs very vulnerable to changes in DNA methylation levels, resulting in frequent changes in their DNA methylation levels. If the first scenario is true, functional selection of DNA methylation changes during tumorigenesis, DNA hypermethylation on a given ICR should be also accompanied with other changes in the neighboring imprinted genes, for instance either hyper or hypomethylation on the nearby imprinted genes. However, the DNA hypermethylation patterns observed from ICRs so far appear to be isolated epigenetic abnormalities without the other accompanying DNA methylation changes (Figure 5 and Supplementary Data 7–8). This indicates that the first scenario may not be the case. Thus, we favor the second possibility that the epigenetic instability observed from ICRs is mostly reflecting the unusual properties associated with the genomic sequences of ICRs. If this is indeed the case, several ICRs reported in this study may be an excellent epigenetic biomarker for cancer detection.

Another interesting observation was derived from the DNA methylation levels of several ECRs within imprinted domains (Figures 6 and 7). The results provide two unexpected aspects of the two ECRs, ECR18 of the PEG3 domain and ECR1 of the H19/IGF2 domain. First, these two ECRs are usually marked with histone modifications, H3K4me1 and H3K27ac, without DNA methylation in the mouse, suggesting active enhancer roles for these two ECRs. Yet, these potential enhancers were found to be methylated in the normal tissues of humans. This is quite unexpected given the global and ubiquitous roles that may be played by these ECRs for the corresponding imprinted domains. Second, the DNA methylation levels of these ECRs appear to be very variable between different tissues and also among individuals. The observed variability might be an indication that the DNA methylation levels of these ECRs are dynamically fluctuating in normal cells depending upon their functional roles for the nearby genes. If this is the case, the DNA methylation levels of these ECRs should not be a good biomarker detecting human cancers. Nevertheless, it should be interesting to investigate the dynamic nature of the DNA methylation levels of these ECRs since it might provide many uncharacterized aspects of the transcriptional control governing each imprinted domain.

## Supplementary Material

SUPPLEMENTARY DATA
