# Supplementary material for: Epigenetic instability of imprinted genes in human cancers
Source: Nucleic Acids Res. 2015 Sep 3;43(22):10689–99. doi: 10.1093/nar/gkv867 (PMC4678850; doi:10.1093/nar/gkv867)
Supplement: SUPPLEMENTARY DATA [file supp_43_22_10689__index.html]

Epigenetic instability of imprinted genes in human cancers — SUPPLEMENTARY DATA 

# Epigenetic instability of imprinted genes in human cancers

## SUPPLEMENTARY DATA

- SUPPLEMENTARY DATA
- SUPPLEMENTARY DATA
- SUPPLEMENTARY DATA
- SUPPLEMENTARY DATA
- SUPPLEMENTARY DATA
- SUPPLEMENTARY DATA
- SUPPLEMENTARY DATA
- SUPPLEMENTARY DATA
- SUPPLEMENTARY DATA
- SUPPLEMENTARY DATA
- SUPPLEMENTARY DATA
- SUPPLEMENTARY DATA
- SUPPLEMENTARY DATA
- SUPPLEMENTARY DATA
- SUPPLEMENTARY DATA
- SUPPLEMENTARY DATA
- SUPPLEMENTARY DATA
- SUPPLEMENTARY DATA
- SUPPLEMENTARY DATA
