# Supplementary material for: Epigenetic instability of imprinted genes in human cancers
Source: Nucleic Acids Res. 2015 Sep 3;43(22):10689–99. doi: 10.1093/nar/gkv867 (PMC4678850; doi:10.1093/nar/gkv867)
Supplement: SUPPLEMENTARY DATA [file supp_gkv867_nar-01327-h-2015-File003.docx]

**Supplemental Data**

**Supplemental Data 1**. A table summarizing the down- and up-regulation of the imprinted gene set in TCGA database. This summary table has been used for generating the image in Figure 1A.

**Supplemental Data 2**. A table summarizing the hypo- and hypermethylation of the imprinted gene set in TCGA database. This summary table has been used for generating the image in Figure 2A.

**Supplemental Data 3**. A table summarizing the down- and up-regulation of the cancer gene set in TCGA database. This summary table has been used for generating the image in Figure 3A.

**Supplemental Data 4**. A table summarizing the hypo- and hypermethylation of the cancer gene set in TCGA database. This summary table has been used for generating the image in Figure 3B.

**Supplemental Data 5**. A list of oligonucleotides used for the PCR amplification of each genomic region. The sequence and genomic position for each primer have been included along with the size of each PCR product.

**Supplemental Data 6**. Genomic coordinates for the imprinted genes and PANCAN genes. The table includes the genomic positions for each imprinted domain and individual genes.

**Supplemental Data 7**. A set of compiled COBRA results derived from the DNA panel of normal and cancer samples.

**Supplemental Data 8**. A set of compiled COBRA results derived from the DNA panel of two sets of paired samples (breast and lung).

**Supplemental Data 9**. A set of DNA methylation results derived from NGS-based sequencing of the PCR products amplified from the DNA panel of normal and cancer samples. For each locus, the methylation level of each sample is presented with the number of sequence reads used for calculating the methylation level.
