# Supplementary figures and images for: Epigenetic instability of imprinted genes in human cancers
Source: Nucleic Acids Res. 2015 Sep 3;43(22):10689–99. doi: 10.1093/nar/gkv867 (PMC4678850; doi:10.1093/nar/gkv867)

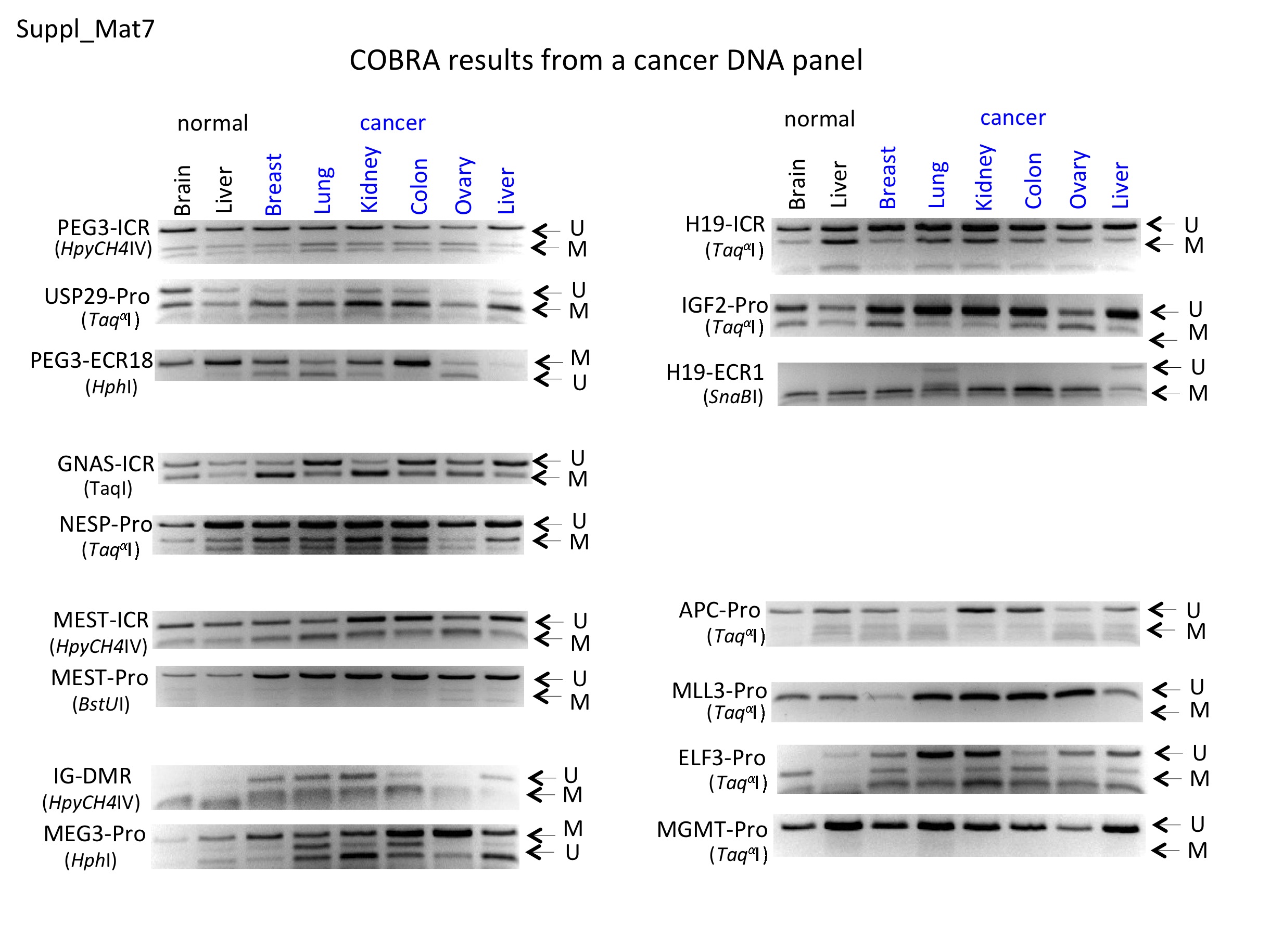

Supplement: SUPPLEMENTARY DATA [file supp_gkv867_nar-01327-h-2015-File010.jpg]

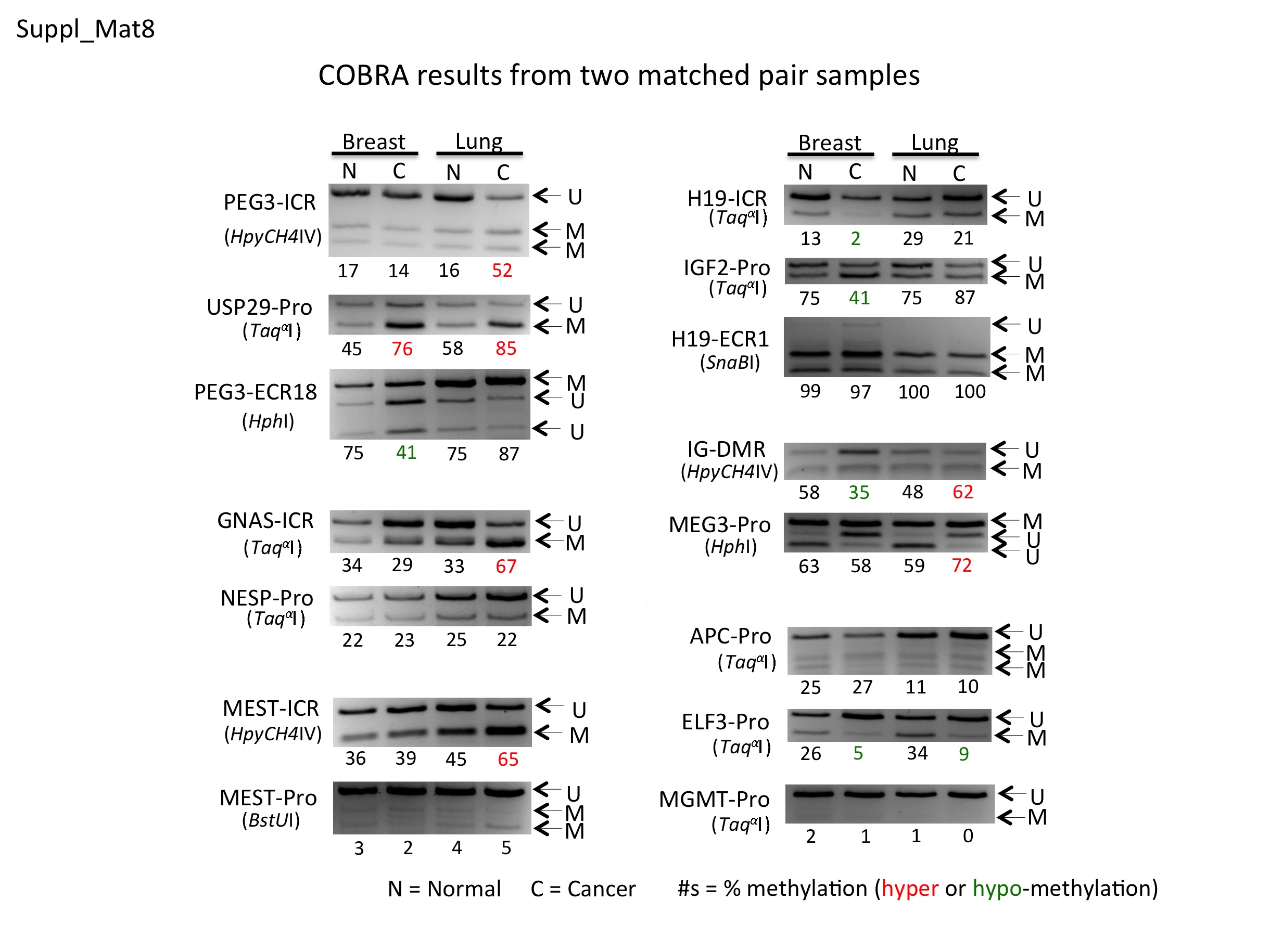

Supplement: SUPPLEMENTARY DATA [file supp_gkv867_nar-01327-h-2015-File011.jpg]

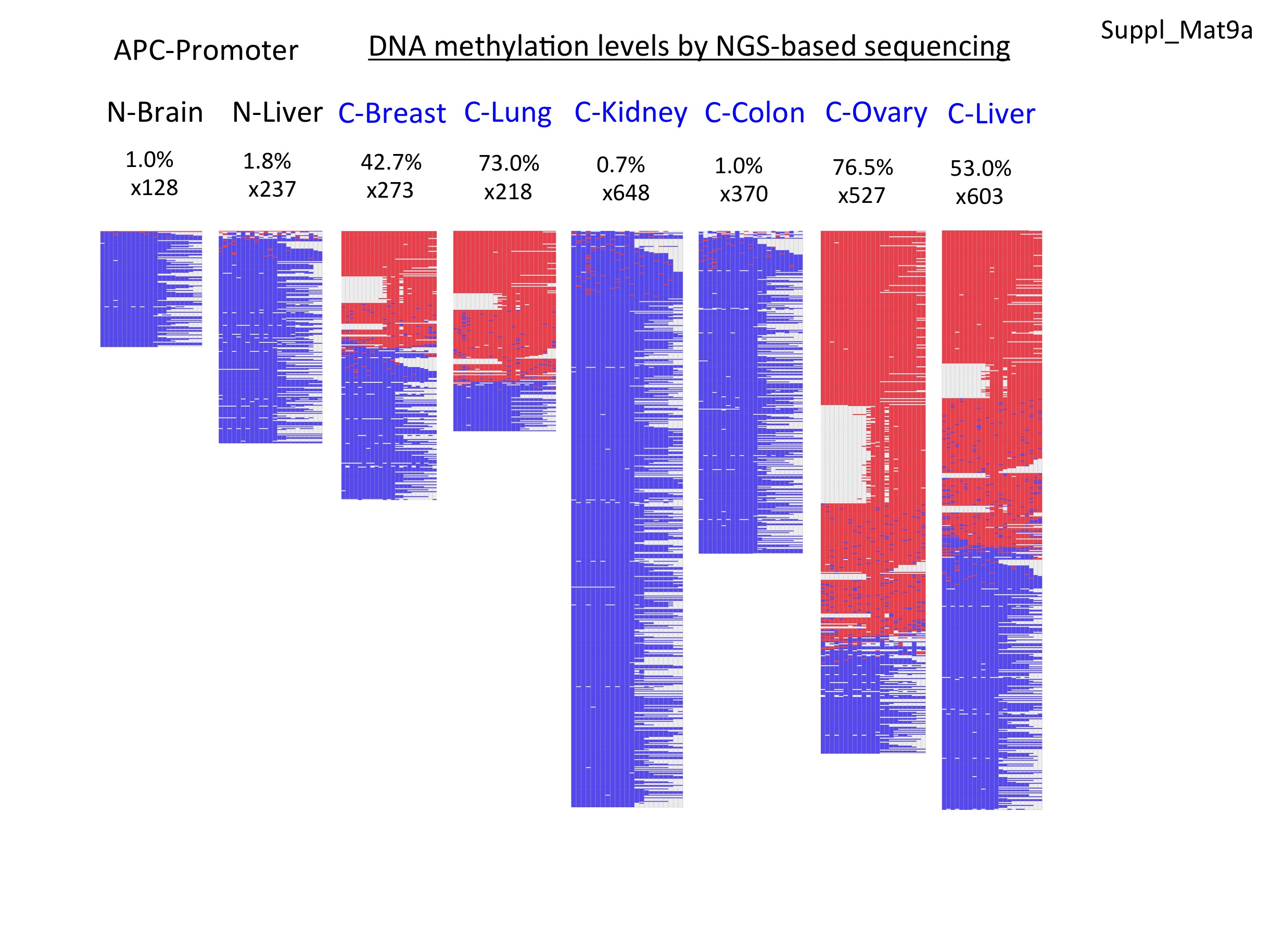

Supplement: SUPPLEMENTARY DATA [file supp_gkv867_nar-01327-h-2015-File012.jpg]

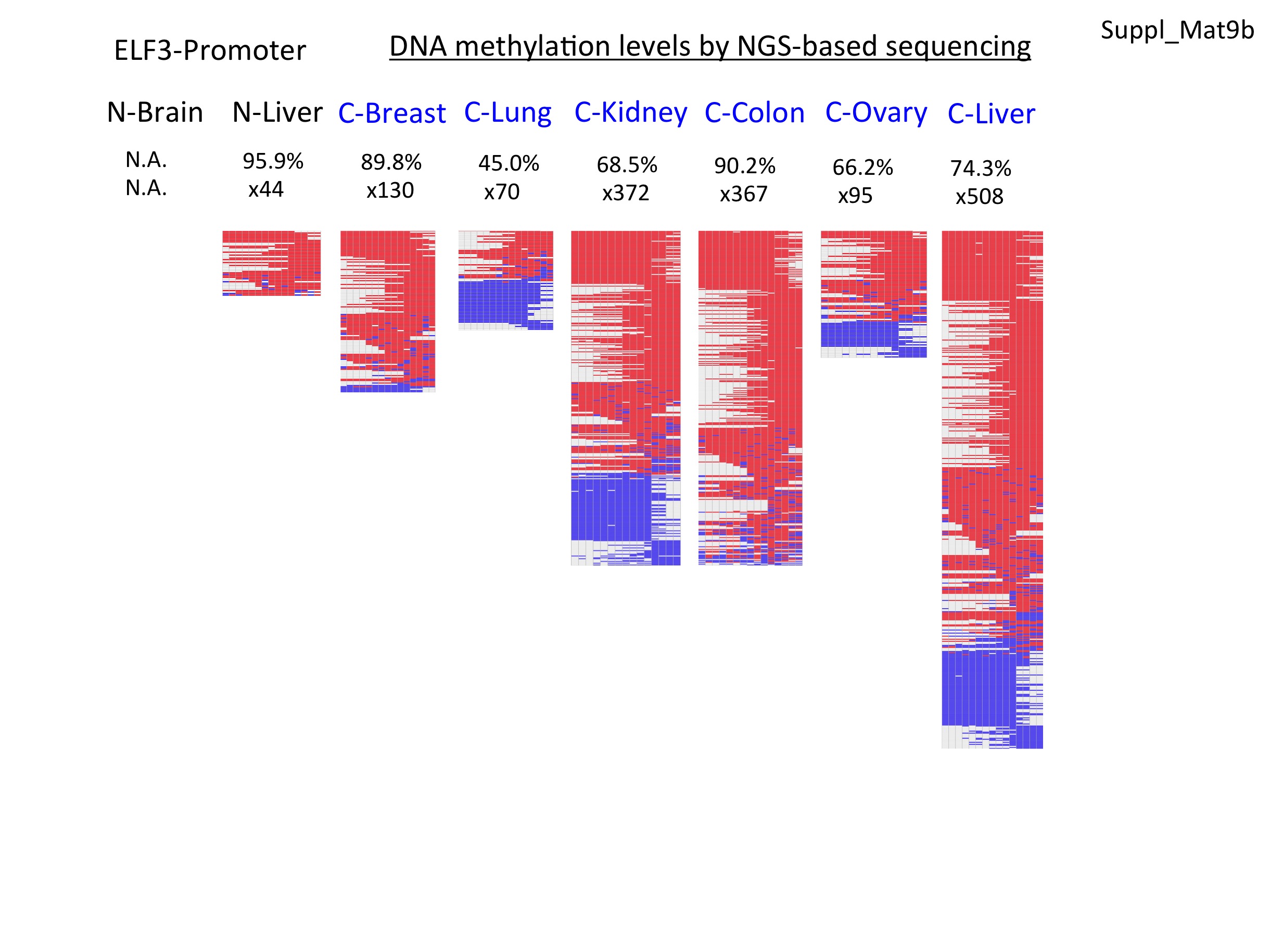

Supplement: SUPPLEMENTARY DATA [file supp_gkv867_nar-01327-h-2015-File013.jpg]

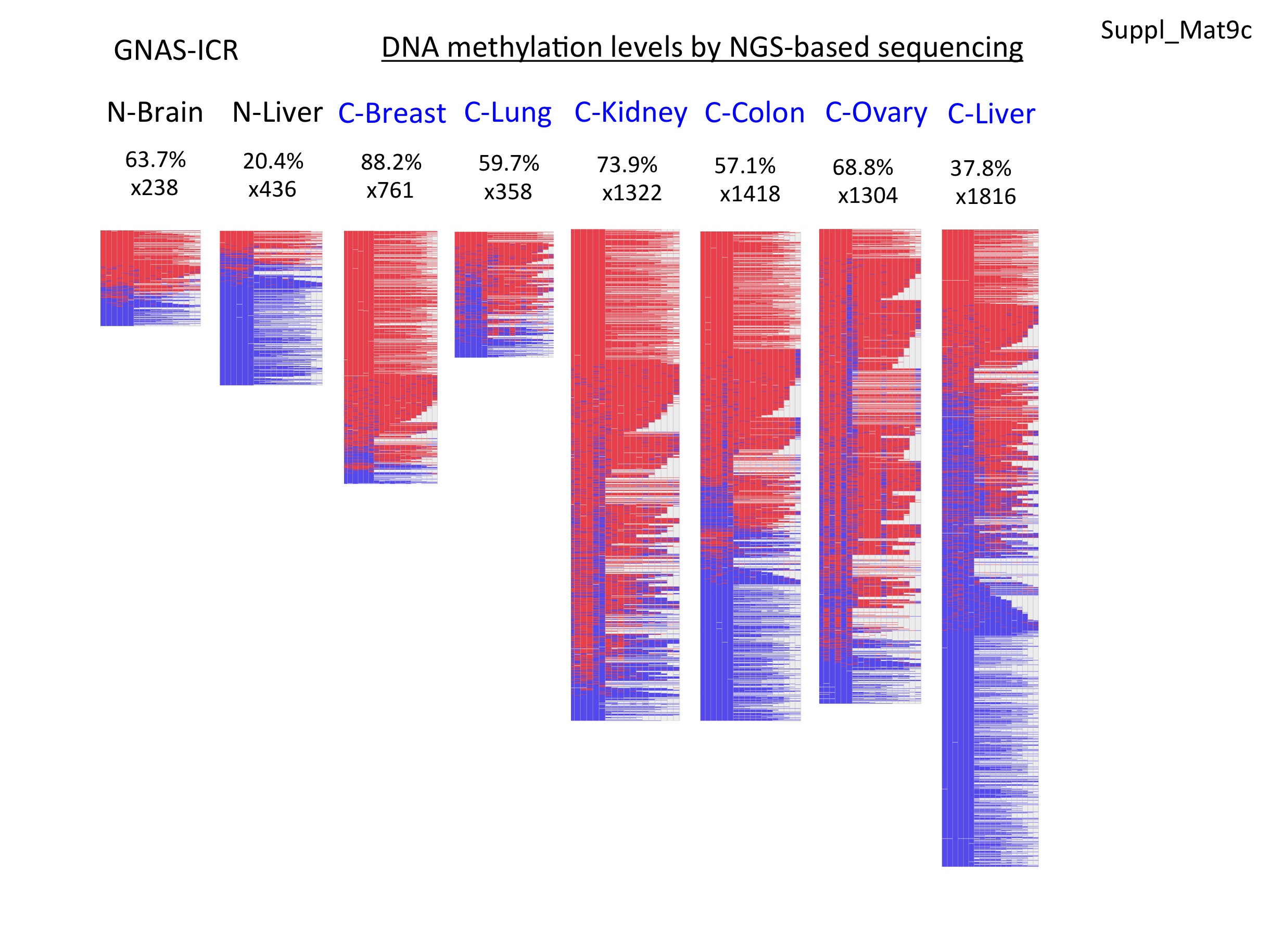

Supplement: SUPPLEMENTARY DATA [file supp_gkv867_nar-01327-h-2015-File014.jpg]

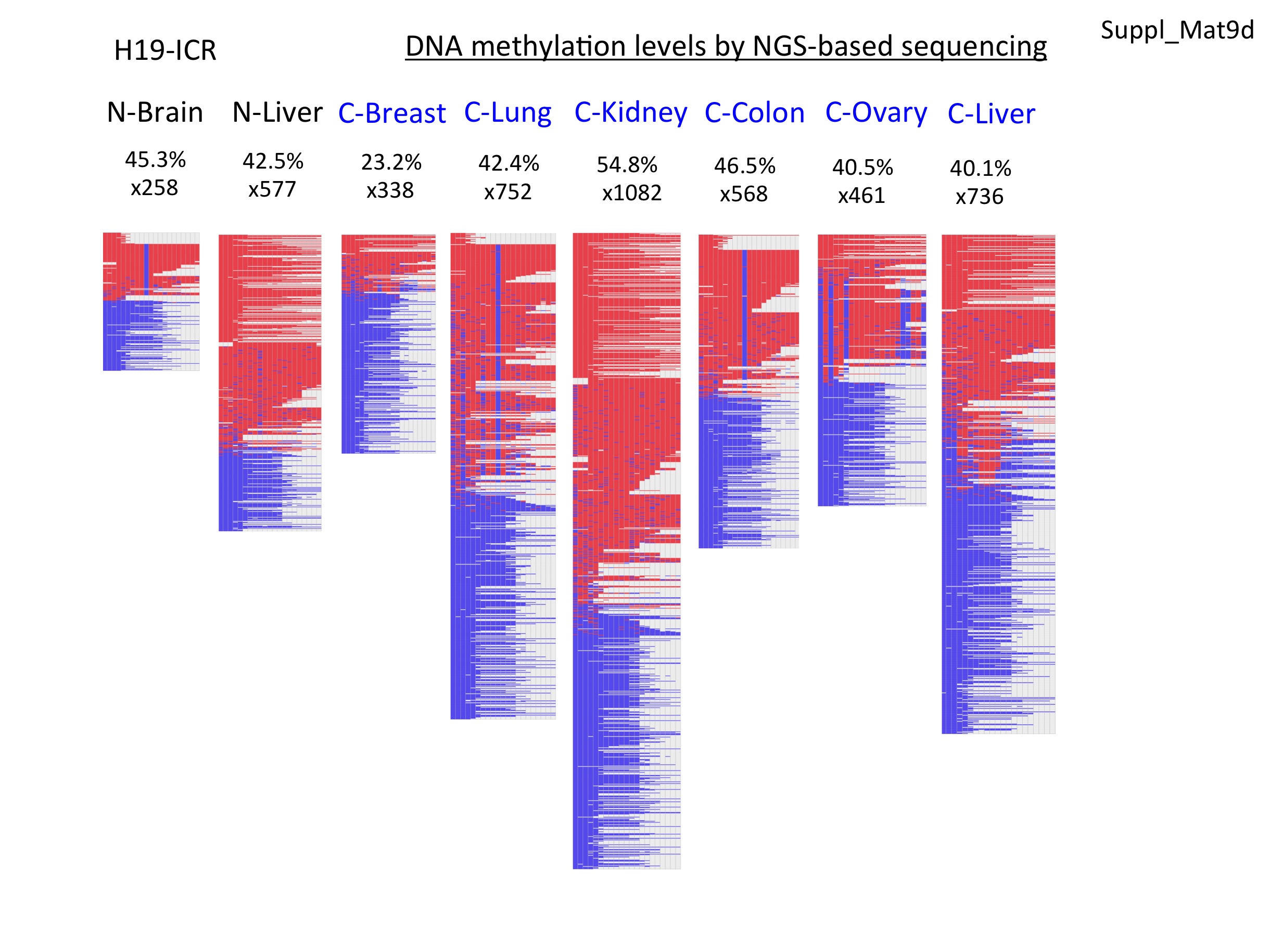

Supplement: SUPPLEMENTARY DATA [file supp_gkv867_nar-01327-h-2015-File015.jpg]

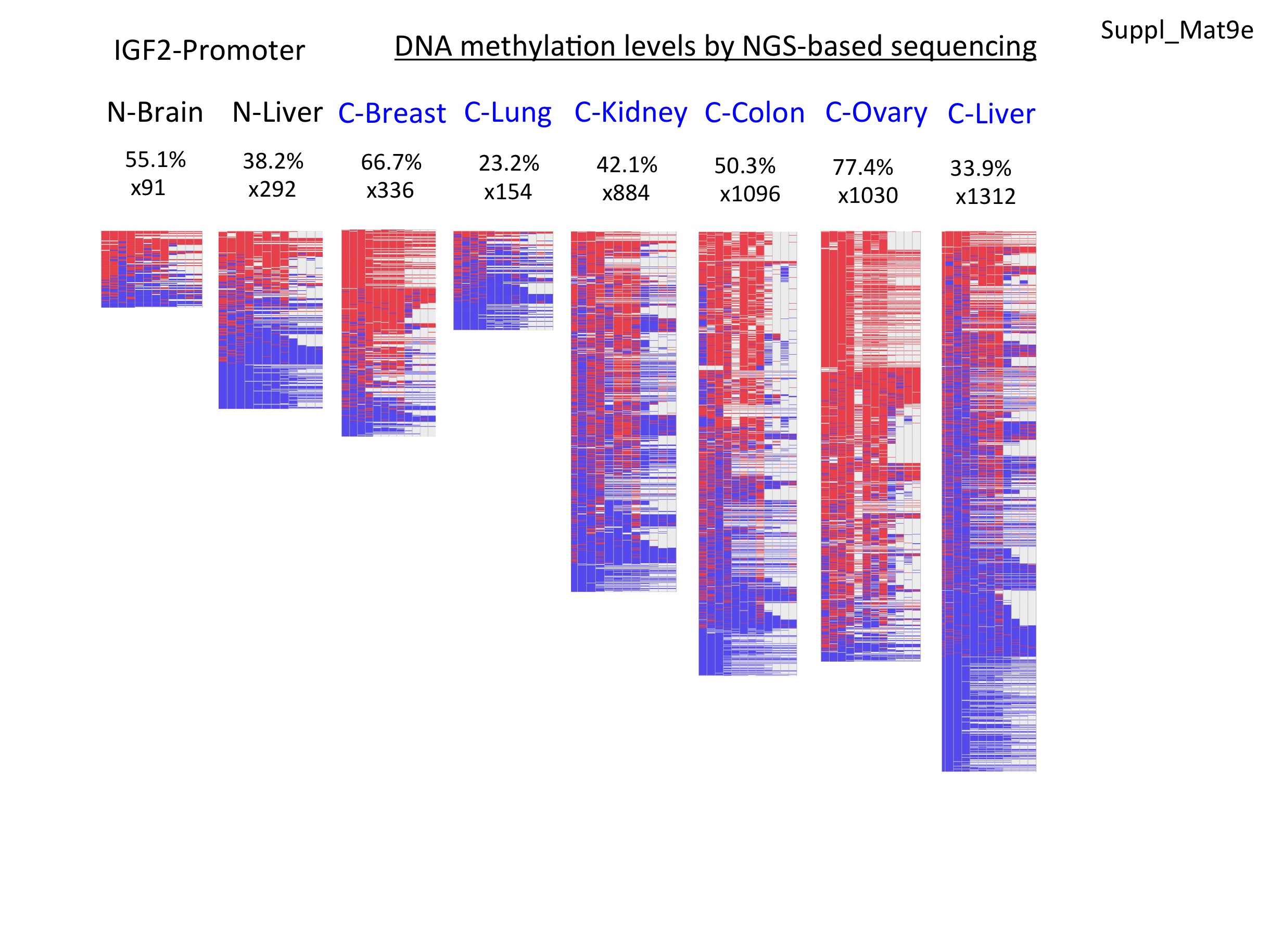

Supplement: SUPPLEMENTARY DATA [file supp_gkv867_nar-01327-h-2015-File016.jpg]

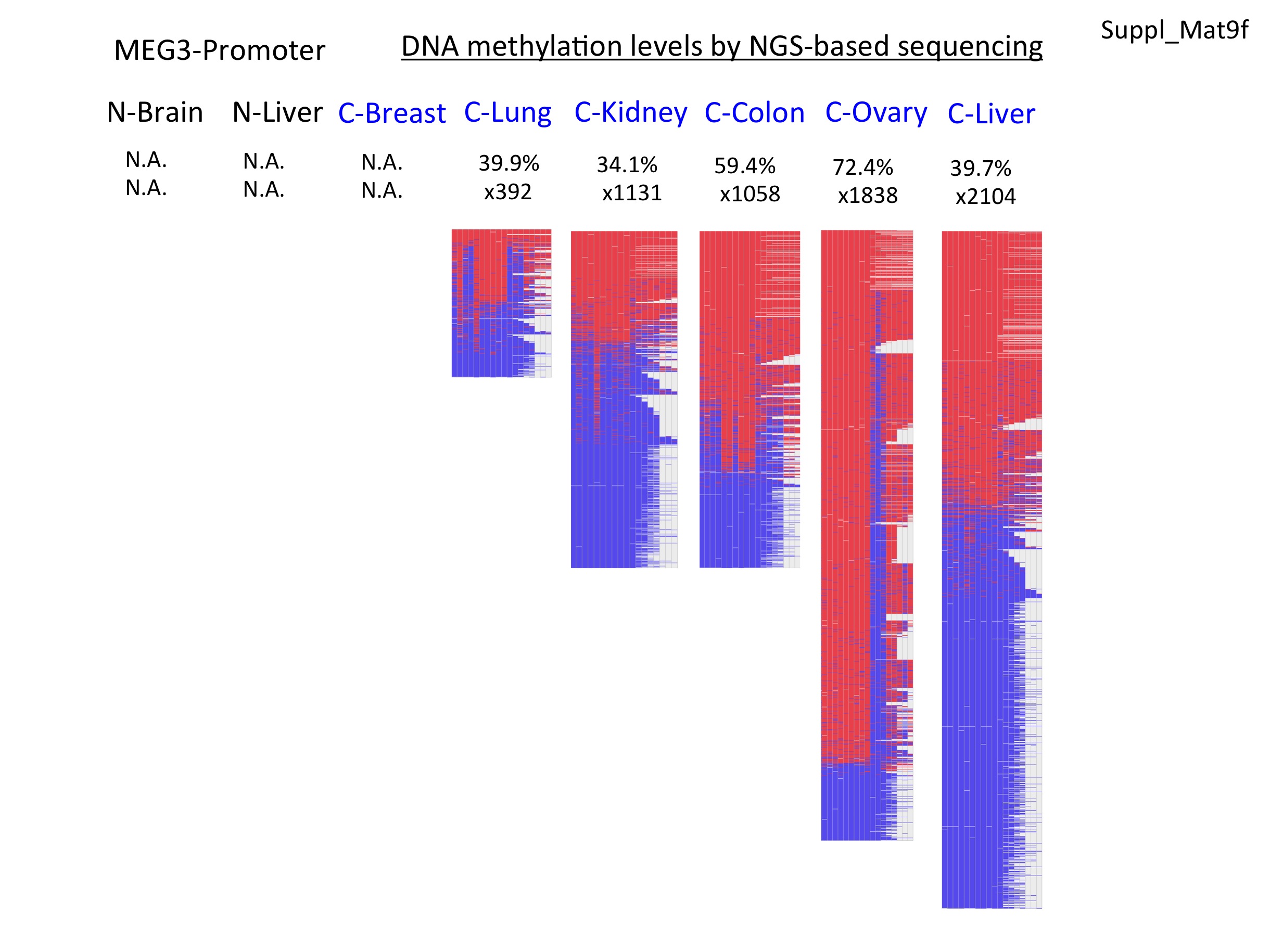

Supplement: SUPPLEMENTARY DATA [file supp_gkv867_nar-01327-h-2015-File017.jpg]

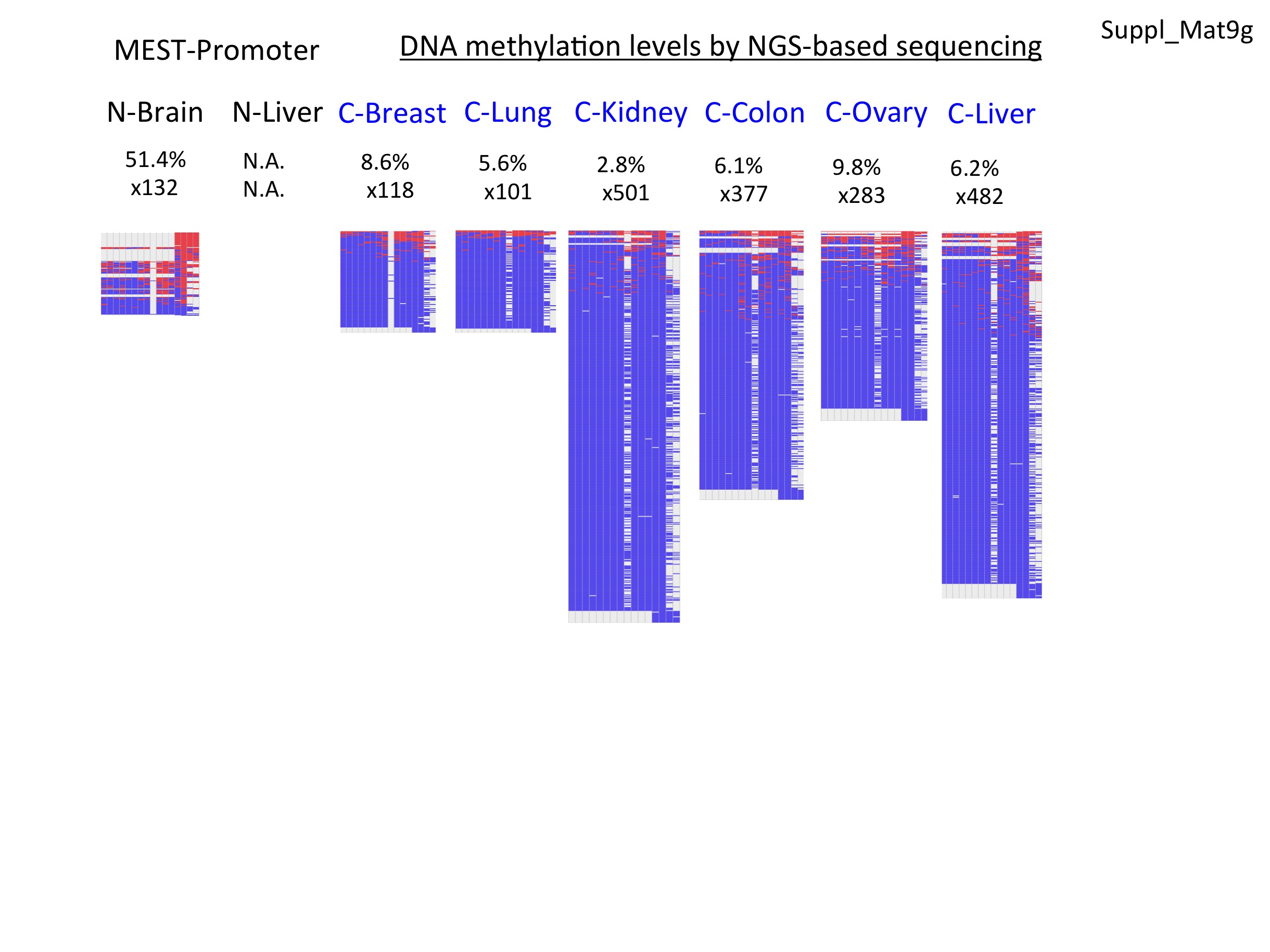

Supplement: SUPPLEMENTARY DATA [file supp_gkv867_nar-01327-h-2015-File018.jpg]

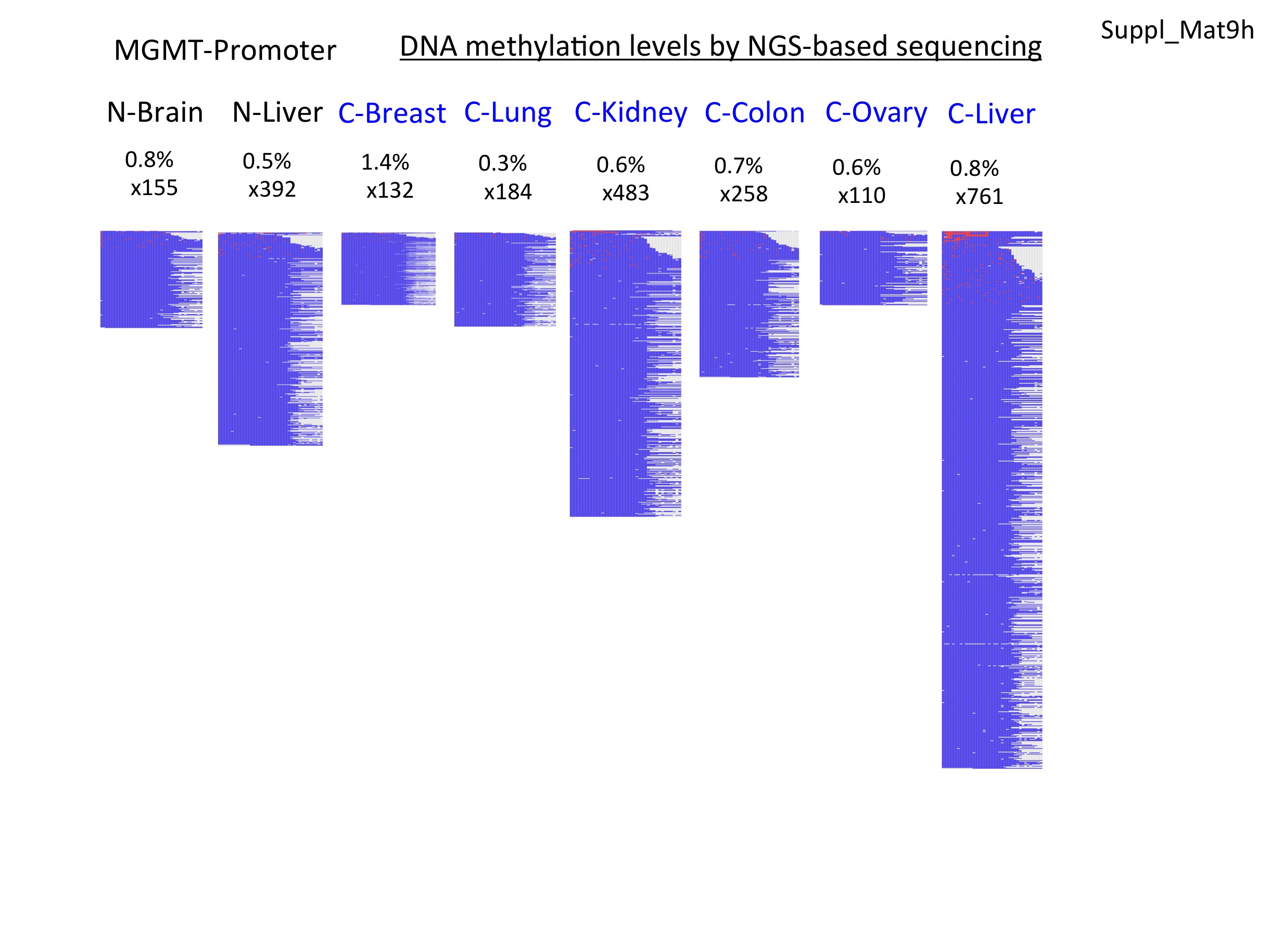

Supplement: SUPPLEMENTARY DATA [file supp_gkv867_nar-01327-h-2015-File019.jpg]

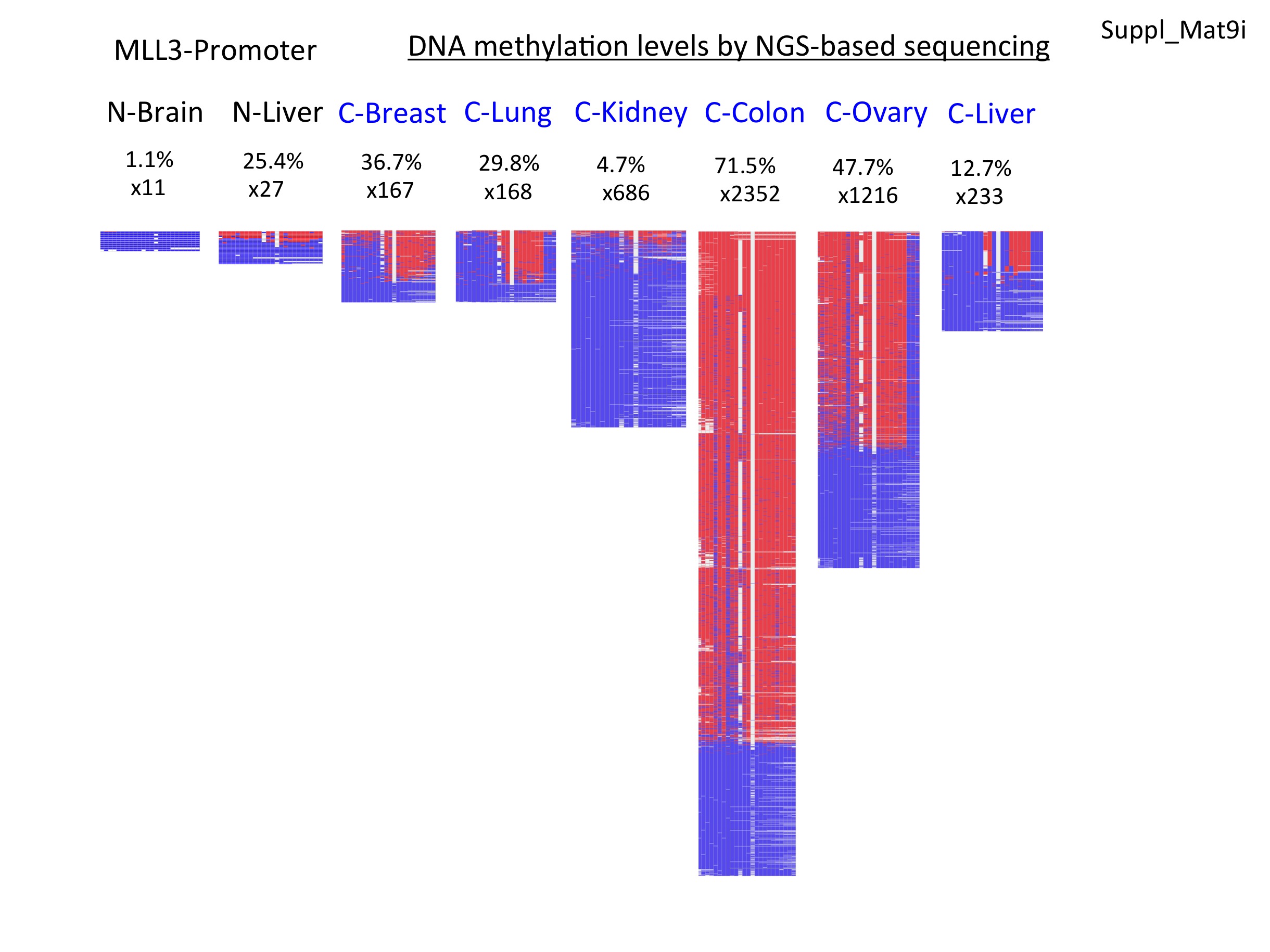

Supplement: SUPPLEMENTARY DATA [file supp_gkv867_nar-01327-h-2015-File020.jpg]

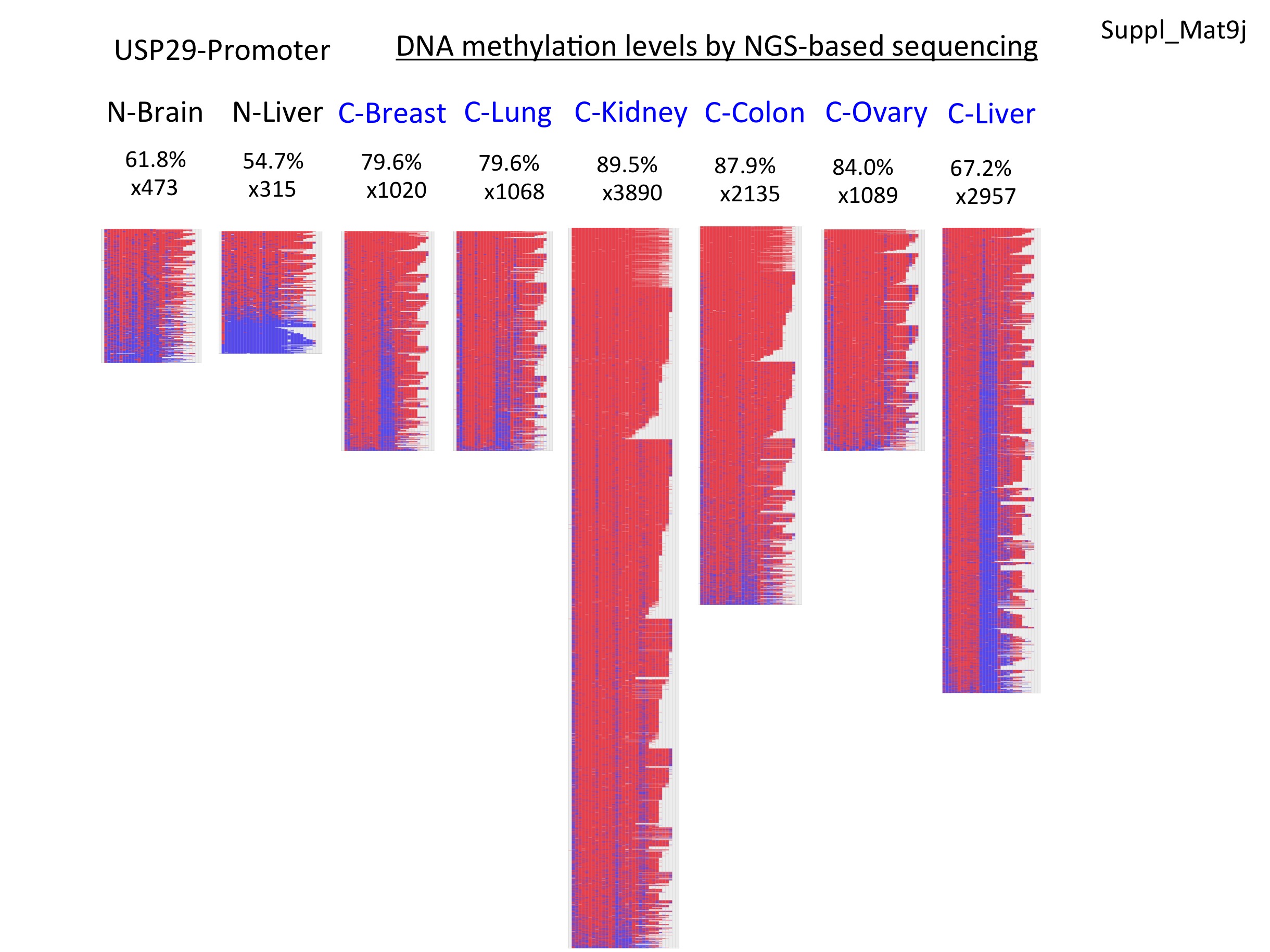

Supplement: SUPPLEMENTARY DATA [file supp_gkv867_nar-01327-h-2015-File021.jpg]
